# Effects of an ICT-Based Wearable Intervention on Physical Function in Arteriosclerosis Obliterans: A 12-Week Study

**DOI:** 10.3390/life16030441

**Published:** 2026-03-09

**Authors:** Gwon-Min Kim, Jaewon Choi, Changsung Han, Miju Bae, Jong-Hwan Park, Il Jae Wang, Bokun Kim, Chanhee Song, Up Huh

**Affiliations:** 1 Medical Research Institute, Pusan National University School of Medicine, Yangsan 50612, Republic of Korea; rlarnjsals47@gmail.com (G.-M.K.); cjw4783@gmail.com (J.C.); chsong0125@pusan.ac.kr (C.S.); 2 Department of Thoracic and Cardiovascular Surgery, Pusan National University School of Medicine, Biomedical Research Institute, Pusan National University Hospital, Busan 49241, Republic of Korea; hcs7717@naver.com (C.H.); nabikr@naver.com (M.B.); 3 Department of Convergence Medicine, Pusan National University School of Medicine, Yangsan 50612, Republic of Korea; parkj@pusan.ac.kr; 4 Department of Clinical Bio-Convergence, Graduate School of Convergence in Biomedical Science, Pusan National University School of Medicine, Yangsan 50612, Republic of Korea; 5 Convergence Medical Institute of Technology, Pusan National University Hospital, Busan 49241, Republic of Korea; 6 Department of Emergency Medicine, Pusan National University School of Medicine, Biomedical Research Institute, Pusan National University Hospital, Busan 49241, Republic of Korea; jrmr9933@gmail.com; 7 Sport Science Innovation Institute, Dongguk University, Seoul 04620, Republic of Korea; fabulousbobo79@gmail.com

**Keywords:** arteriosclerosis obliterans, gait speed, 6 min walk test, wearable device, information and communication technology, moderate-to-vigorous physical activity

## Abstract

Arteriosclerosis obliterans (ASO) is associated with impaired walking function and claudication. However, the effects of information and communication technology (ICT)-based wearable interventions on objectively measured gait outcomes in this population have not been determined. In this 12-week intervention, 52 patients with ASO were randomly assigned to an ICT-based wearable-assisted exercise intervention (*n* = 30) or a control (*n* = 22) group. All participants wore a triaxial accelerometer–based device on the non-dominant wrist to monitor moderate-to-vigorous physical activity (MVPA), expressed as average min/day. The intervention group received structured exercise guidance, including walking and lower-limb strengthening exercises, and weekly feedback based on device data; the control group received no exercise instruction or feedback. Primary outcomes were gait speed and 6 min walk test (6MWT) distance; secondary outcomes included MVPA and cognitive function. The intervention group showed significant improvements in gait speed and 6MWT distance compared with those in the control group (*p* < 0.05), indicating enhanced ambulatory function. An exploratory machine learning analysis suggested that gait speed and 6MWT distance are informative variables for functional-status characterization. ICT-based wearable interventions may serve as scalable approaches for functional rehabilitation in ASO; larger, longer-term studies should confirm these effects and clarify the underlying mechanisms.

## 1. Introduction

Arteriosclerosis obliterans (ASO) is a chronic progressive vascular disease characterized by atherosclerotic narrowing or occlusion of the arteries supplying blood to the lower extremities. ASO is often considered a prototypical condition associated with vascular aging, with a documented prevalence exceeding 20% among individuals aged ≥65 years. Empirical studies have established a strong positive correlation between aging and ASO incidence.

Claudication, a characteristic manifestation of ASO, results from diminished blood flow due to arterial constriction. This condition commonly leads to musculoskeletal discomfort in the lower limbs during physical engagement, which generally resolves within approximately 10 min of exertion cessation [1]. ASO is recognized as the primary causal agent for intermittent claudication and is indicative of significant peripheral artery disease (PAD) [2,3]. Claudication is correlated with slow deterioration in physical performance, negatively influencing gait speed, exercise endurance, and muscle contraction frequency, among other parameters [4]. The chronic physical activity limitation experienced by patients with ASO results in measurable alterations in gait characteristics. Previous studies have shown that intermittent claudication is associated with reduced walking speed, shortened step length, and impaired walking endurance [5]. These biomechanical alterations not only impair functional mobility but also increase fall risk and musculoskeletal complications, further reducing overall quality of life and exercise capacity. Accordingly, objective assessment of walking performance is essential for evaluating functional status in this population. Reduced gait speed and decreased walking endurance, often quantified using the 6 min walk test (6MWT), are considered key clinical indicators of functional limitation and disease severity in patients with ASO and PAD. Consequently, individuals with ASO often exhibit lower performance on functional assessments than that of their healthy older counterparts. In addition to physical restrictions, ASO detrimentally affects the overall quality of life and increases the probability of cardiovascular complications.

Engaging in a daily routine involving moderate-to-vigorous physical activities (MVPAs) for a minimum of 30 min has been thoroughly substantiated as a viable strategy for preventing and managing cardiovascular pathologies [6,7]. Supervised exercise interventions are notably effective in alleviating symptoms and improving the functional capacity of patients with PAD and intermittent claudication [8]. Recent progress in information and communication technology (ICT) has enabled the immediate evaluation of exercise performance using wearable sensor technology [9]. ICT-based systems offer avenues for customized exercise evaluations, progress surveillance, and dynamic alterations of training programs, thereby augmenting the efficacy of and adherence to rehabilitation methodologies in patients with ASO.

Despite the growing application of wearable devices and ICT-based interventions in rehabilitation, evidence remains limited regarding their effectiveness in improving walking function in patients with claudication secondary to ASO. In particular, studies of objectively measured gait-related outcomes, such as gait speed and the 6MWT, are lacking in this population [10]. Therefore, further investigation is warranted to determine whether an ICT-based wearable-assisted exercise intervention can effectively improve these key functional outcomes in patients with ASO.

Academic research focused on the enhancement of claudication gait using ICT-integrated wearable monitoring interventions is remarkably limited. In this study, the effects of an ICT-based wearable–assisted exercise intervention on physical function in individuals with ASO were evaluated, with specific emphasis on gait speed and performance on the 6MWT. Furthermore, an exploratory machine learning approach was applied, not as a primary predictive model but as a supplementary analysis to examine the relative importance of physical function-related variables in this study population [11].

## 2. Materials and Methods

### 2.1. Study Design and Participants

**Figure 1:**
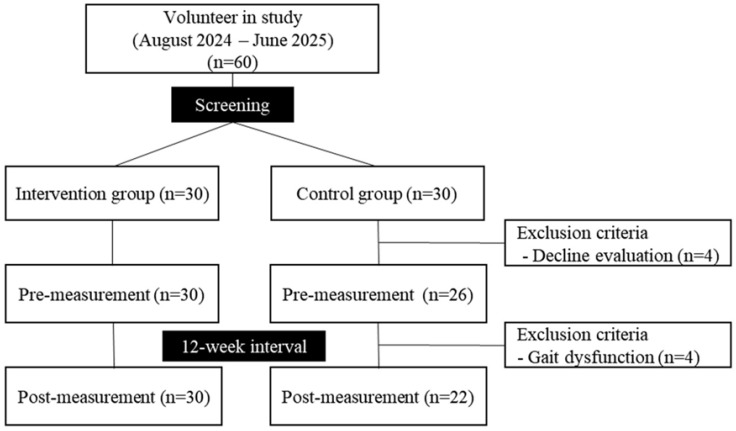
Flow chart of study participants.

Individuals were systematically recruited from August 2024 to June 2025 (Figure 1). After eligibility screening, participants were randomly assigned in a 1:1 ratio to either the intervention or control group (*n* = 30 per group). (1) the random allocation sequence was generated using computer-generated random numbers, and (2) the allocation was implemented by an independent third party not involved in study conduct, thereby reducing the risk of allocation-related bias. During the study period, eight participants in the control group were excluded; four who declined further evaluation and four owing to gait dysfunction. Consequently, data for 52 participants (30 in the intervention group and 22 in the control group) were included in the final analysis. We also clarified that our main analysis corresponds to a per-protocol (PP) approach (i.e., participants who completed follow-up assessments).

This study was approved by the Research Ethics Committee of the Pusan National University Hospital (2408-002-141). All participants were provided with a comprehensive overview of the study protocol, and written informed consent was obtained prior to their participation in accordance with the ethical principles delineated in the Declaration of Helsinki. All methodologies strictly followed the sanctioned study protocol as well as pertinent guidelines and regulations.

### 2.2. Intervention Program

**Figure 2:**
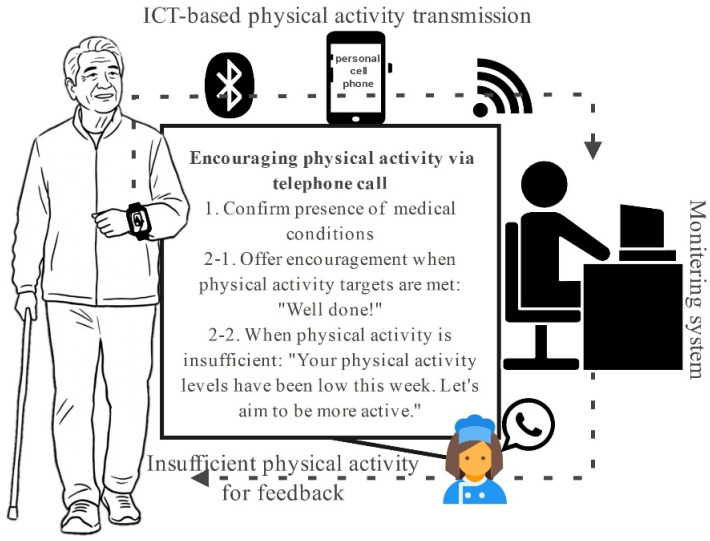
Schematic diagram of a remote physical activity monitoring system based on wearable sensors.

This study utilized an ICT-based wearable monitoring system as the primary tool for quantifying physical activity, combined with a structured exercise intervention program designed for patients with ASO. Physical activity levels were quantified objectively throughout the study period using a triaxial accelerometer–based wearable device (Fitmeter, Suwon, Republic of Korea). The device was worn on the non-dominant wrist during waking hours and continuously recorded acceleration data, which were subsequently converted into estimates of energy expenditure expressed as metabolic equivalents (METs). moderate-to-vigorous physical activity (MVPA) was defined as activity exceeding 3 METs. Participants were instructed to wear the device for at least 10 h per day, and a valid monitoring period was defined as a minimum of 4 days per week, including at least 1 weekend day. The device was removed only during water-based activities, such as bathing or showering. Participants in the intervention group were encouraged to engage in at least 30 min of MVPA per day, with a gradual progression toward a target of 45–60 min as tolerated, based on the Frequency, Intensity, Time, and Type principle. The primary recommended activity was walking, performed either outdoors or in an indoor walking environment, in accordance with the provided exercise guideline. In addition, participants in the intervention group received a structured exercise program guidelines involving approximately 15 min of warm-up exercises, including neck stretching, trunk rotation, and leg-raising movements; lower-limb strengthening exercises, including calf pump exercises (40 repetitions); and moderate-intensity walking for at least 20 min. To support adherence, a research nurse reviewed activity data and provided weekly telephone calls or text messages to participants in the intervention group, particularly when the prescribed activity goals were not achieved. Follows: 1. Confirm the presence of any medical conditions. 2-1. Provide positive reinforcement when physical activity targets are achieved, such as stating, “Well done!” 2-2. If physical activity is deemed insufficient, communicate, “Your physical activity levels have been low this week. Let us aim to be more active.” The control group also wore the same device for activity monitoring; however, they did not receive exercise instruction, supervision, or feedback during the study period (Figure 2).

### 2.3. Clinical Measurements

The diagnosis of ASO was substantiated by the manifestation of ischemic symptoms in the lower extremities, including intermittent claudication, in conjunction with an ankle–brachial index of ≤0.90 based on prevailing international directives [12].

### 2.4. Physical Function Measurements

Grip strength was quantified in kg using a handgrip dynamometer (TKK-5401; Takei Scientific Instruments, Tokyo, Japan) strategically placed on the participants’ dominant hands. During assessment, the participants were required to maintain an upright posture with their feet positioned shoulder-width apart and their elbows fully extended while maintaining a forward gaze [13]. The 6-Minute walk test (6MWT) was conducted on a stable level surface in an outdoor environment, with the participants walking along a straight 30 m pathway. The participants were instructed to cover the maximum distance achievable within 6 min without engaging in ambulation [14]. The total distance walked was measured to the nearest meter. For gait speed assessment, a 6 m course was established, incorporating segments allocated for acceleration, time trials, and deceleration to minimize bias. The participants were instructed to ambulate at their habitual pace to simulate real-world walking conditions. The assessment commenced with the verbal cue “go” following a 3 s countdown and concluded when the participants reached a distance of 4 m [15]. For the five-time sit-to-stand (FTSS) test, a straight-backed chair was placed adjacent to a wall. The participants were instructed to cross their arms and execute the stand–sit maneuver five times as quickly as possible [16]. Time was measured from the initial command to the moment the participant’s buttocks contacted the seat following the fifth stand, recorded to the nearest hundredth of a second using a digital stopwatch.

### 2.5. Other Measurements

Demographic data were obtained via a structured questionnaire. The body mass index (BMI) and skeletal muscle mass were evaluated using a specialized instrument placed at S10 (InBody Co., Ltd., Seoul, Republic of Korea). All measurements were conducted with participants in a seated or supine position following the manufacturer’s standardized protocol. Depressive symptoms were identified using the Short-Form Geriatric Depression Scale translated into Korean (SGDS-K) [17]. Each item is scored as 0 or 1, yielding a total score ranging from 0 to 15, with higher scores indicating greater depressive symptomatology. The Korean version of the Mini-Mental State Examination (K-MMSE) was used for cognitive decline assessment [18]. Scores range from 0 to 30, with higher scores reflecting better cognitive performance. Health-related quality of life was measured using the World Health Organization Quality of Life–BREF Korean version (WHOQOL-BREF) [19]. Each item is rated on a 5-point Likert scale (1–5), and domain scores are transformed to a 0–100 scale, with higher scores denoting better quality of life. Throughout the administration of the SGDS-K, K-MMSE and WHOQO, the investigator clearly articulated each item, facilitating participant responses.

### 2.6. Statistical Analysis

Statistical analyses were performed using SPSS 27 (IBM Corp., Armonk, NY, USA). The characteristics of the study population are presented as means ± standard deviations (SD) for continuous variables and as frequencies and proportions for categorical variables. Normality of the data distribution was assessed using the Shapiro–Wilk test, and homogeneity of variances was examined using Levene’s test. The chi-squared test was used for hypothesis testing when appropriate. Correlations between two variables were evaluated using Pearson’s correlation coefficient. With respect to machine learning methodologies, a random forest analysis was conducted in an exploratory manner to examine the relative importance of physical function-related variables within the study population. Feature importance was estimated using a permutation-importance approach. No independent validation dataset or cross-validation procedure was applied owing to the limited study sample; therefore, the results of the machine learning analysis should be interpreted with caution, with the potential risk of overfitting. Analytical processes were performed using Python 3.13.5 on a Windows operating system. Two-way repeated-measures analysis of variance was used to investigate the mean variances linked to the interactions between temporal factors and group categorization. Post hoc pairwise comparisons with Bonferroni correction were performed for significant interactions. Statistical significance was set at a *p*-value of <0.05.

## 3. Results

Table 1 summarizes the demographic data for the participants. The mean participant age was 68 years, with a non-significant age disparity between the intervention (67 years) and control (70 years) groups (*p* = 0.224). Male participants accounted for approximately 48% (28 men and 20 women, *p* = 1.000). The average height and weight were 169 cm and 66 kg, respectively, yielding a BMI of 23 kg/m^2^. The mean skeletal muscle mass was 27 kg. No significant differences in body metrics were observed between the groups (all *p* > 0.05). The average systolic and diastolic blood pressures were 129 and 67 mmHg, respectively, with higher diastolic blood pressure measurements in the intervention group than in the control group (72 vs. 61 mmHg, *p* = 0.011). Calf circumference was consistent for both legs in each group, with an average of 36 cm. Hypertension, diabetes, and hypercholesterolemia were detected in 58%, 35%, and 46% of the participants, respectively. Approximately 12% of the participants were alcohol drinkers, and 12% were smokers, with no significant between-group differences (*p* = 0.051 for both). The prevalence of hypertension was higher (60% vs. 55%) and the prevalence of diabetes was lower (27% vs. 45%) in the intervention group than in the control group; however, the differences were not significant (*p* = 0.078 and *p* = 0.239). Hypercholesterolemia rates were comparable between the groups (40% vs. 55%, *p* = 0.400).

**Figure 3:**
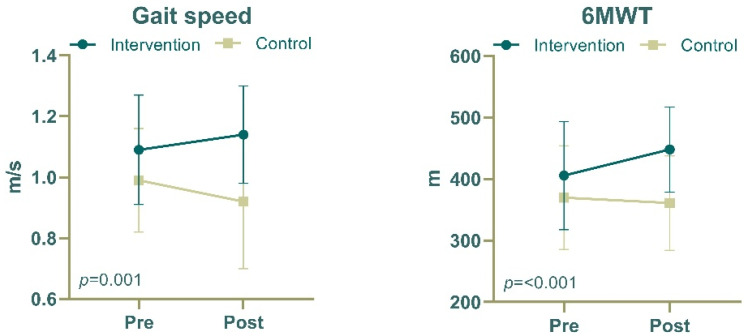
Changes in physical function parameters between the groups after 12 weeks using the adjusted model. Adjustments for age, sex, alcohol consumption, and smoking were made. 6MWT, 6 min walk test.

Table 2 and Figure 3 show the changes in physical and cognitive function parameters between the intervention and control groups before and after the 12-week intervention period. Grip strength improved in both groups, increasing from 30.80 ± 5.87 kg to 32.41 ± 5.16 kg in the intervention group and from 28.06 ± 6.45 kg to 30.45 ± 5.85 kg in the control group; however, no significant between-group differences were observed (*F* = 0.718, *p* = 0.401). Gait speed improved significantly in the intervention group, increasing from 1.09 ± 0.18 m/s to 1.14 ± 0.16 m/s; in contrast, the control group exhibited a slight decrease from 0.99 ± 0.17 m/s to 0.92 ± 0.22 m/s (*F* = 12.615, *p* < 0.001). The 6MWT distance improved significantly in the intervention group; specifically, it increased from 405.70 ± 87.86 m to 448.00 ± 69.07 m in the intervention group but changed slightly from 369.80 ± 84.39 m to 361.00 ± 76.94 m in the control group (*F* = 10.345, *p* = 0.002). Performance on the FTSS test did not differ significantly between the groups (*F* = 0.454, *p* = 0.504), with both groups maintaining similar completion times throughout the study period. The WHOQOL score remained relatively stable in both groups, with no significant between-group differences (*F* = 0.063, *p* = 0.803). The intervention group maintained a stable MMSE score (from 27.80 ± 2.14 to 27.87 ± 2.10), whereas the control group demonstrated a small numerical increase (from 27.18 ± 1.89 to 28.45 ± 1.65), and the difference between groups was significant (*F* = 9.397, *p* = 0.003). The SGDS score showed no significant between-group differences (*F* = 0.661, *p* = 0.420), with both groups exhibiting minimal changes in depression scores. After adjusting for confounding variables, such as age, sex, alcohol consumption, and smoking habits, marked discrepancies were identified in gait speed (*p* = 0.001) and 6MWT distance (*p* < 0.001). The ranking of measured variables in the random forest model identified gait speed as the most significant parameter, based on its impact on accuracy, followed by the 6MWT (Supplementary Figure S1).

## 4. Discussion

This study showed that a 12-week supervised exercise protocol employing an ICT-based wearable device significantly augments gait velocity and performance metrics on the 6MWT among individuals with ASO, indicating an enhanced capacity for ambulation pertinent to claudication. These findings are consistent with the results of a previous study showing that a 6-month home-based exercise regimen integrating smartphone applications with wearable activity monitors led to substantial improvements in the maximum walking distance among patients with PAD [20].

In this intervention study, participants in the intervention group showed an increase in the 6MWT distance from 405.68 ± 89.41 m to 447.97 ± 70.29 m, accompanied by an improvement in gait velocity from 1.09 ± 0.18 m/s to 1.14 ± 0.17 m/s (Table 2 and Figure 3). Similarly, previous studies have reported that supervised exercise improves the 6MWT distance, balance, and overall physical performance in individuals with intermittent claudication [21,22]. In a randomized controlled trial in volving 156 participants diagnosed with ASO, those allocated to a supervised aerobic treadmill regimen exhibited a mean improvement of 35.9 m in the 6MWT distance over a 6-month period, surpassing the performance of 23.5 m [23]. These findings suggest that the efficacy of interventions utilizing ICT-based supervision in ameliorating walking function deficits induced by claudication in individuals with ASO is equivalent to that of traditional supervised interventions. Also, in an exploratory variable importance analysis (see Supplementary Material), gait speed and 6MWT distance were identified as the most informative variables for characterizing functional status.

The significant improvements in 6 min walk distance and gait speed observed in our study may be explained by multiple synergistic physiological adaptations induced by exercise training. These include enhanced collateral circulation and angiogenesis stimulated by repetitive ischemia–reperfusion cycles [24,25], improved endothelial function through increased nitric oxide bioavailability [26], and enhanced skeletal muscle oxidative capacity with increased mitochondrial density and capillary networks [27,28]. Furthermore, the correlation between improved exercise time and minimum calf muscle oxygen saturation (StO_2_) and peak walking time (r = 0.601, *p* = 0.0015) reported previously supports our findings, suggesting that improvements in microvascular function directly contribute to enhanced walking performance in patients with PAD [29]. However, it is important to acknowledge that our study did not directly measure these physiological parameters. The observed functional gains likely reflect these underlying vascular and muscular adaptations as well as the combined effects of structured guidance, individualized monitoring, and motivational support provided during the intervention, which enhanced exercise adherence and training outcomes.

Individuals participating in the study were identified based on their MMSE scores, which fell between 24 and 30 points (i.e., within permissible normative standards). Even with the significant changes noted in the control group, it is important to recognize that for older individuals residing within this normative range (K-MMSE score of 24–30 points), an MMSE score change of no less than 3 points is essential for such shifts to be interpreted as significant for cognitive function [30]. This threshold presents difficulties in interpreting these changes as significant in older individuals categorized within the normative range. Consequently, the relatively brief duration of the intervention and the nature of the program implemented in this investigation are regarded as inadequate for fostering improvements in cognitive functionality among the older population.

However, supervised exercise protocols are frequently limited by practical impediments, including the demand for recurrent hospital visits and financial considerations tied to facility-based services. Wearable devices that employ accelerometers are practical instruments for monitoring habitual physical activity and promoting exercise adherence in clinical populations. Moreover, investigations involving individuals with vascular diseases have demonstrated that interventions supported by wearable technology can yield significant improvements in ambulatory function, corroborating the findings of the present study. These findings indicate the feasibility of interventions utilizing wearable devices to yield comparable therapeutic outcomes while alleviating the financial strain associated with conventional hospital-supervised therapies. In this study, the increase in MVPA was not statistically significant (*p* = 0.103). This might be due to a small number of participants, which makes it hard to see moderate changes, and because people had different activity levels. Still, most participants kept up with their daily MVPA goals during the study, showing they followed the activity plan well. Since the change in MVPA was not significant, the improvements seen in the study should be viewed carefully. They might not be just because of more physical activity. Other parts of the program, like feedback, personal coaching, and support, might have helped improve gait [31]. Future studies with more participants are needed to understand how these programs work. At the start, there was a significant difference in diastolic blood pressure between the groups, but both were in normal ranges. There was no significant difference in systolic blood pressure or high blood pressure rates. Gait speed and 6 min walk test (6MWT) results show overall fitness, not just resting diastolic blood pressure. So, even though other factors might play a role, it is unlikely that the initial blood pressure difference greatly affected the walking improvements [32].

Several study limitations should be considered. First, the 12-week intervention period did not allow for evaluations of the long-term sustainability of the observed effects or potential rebound phenomena. Second, although both groups wore the same device, the intervention was intentionally designed to include additional supervision and encouragement by a research nurse. Therefore, the effects observed in this study reflect a combined ICT- and human-supported intervention, and the independent effect of the wearable device alone could not be determined. Third, differential attrition occurred in the control group, with several participants withdrawing due to worsening walking difficulty or physical discomfort related to their underlying disease. Although these withdrawals were not related to the intervention itself and occurred after randomization, the possibility of attrition bias cannot be completely excluded. Fourth, baseline levels of habitual physical activity, exercise-related behaviors, and familiarity with digital technologies were not formally assessed. Finally, this was a single-center study with a relatively small sample size, and the machine learning analysis should be regarded as exploratory.

## 5. Conclusions

In summary, an ICT-based wearable-assisted exercise intervention significantly improved gait speed and 6MWT performance in patients with ASO, resulting in enhanced ambulatory function and potential reduction in claudication-related limitations. These findings suggest that ICT-based wearable interventions provide a feasible and scalable approach to supporting functional rehabilitation in individuals with ASO. Further large-scale and long-term studies are warranted to confirm these effects and to clarify the underlying mechanisms.

## Figures and Tables

**Table 1:** Baseline characteristics of the participants.

Variables	Overall (*n* = 52)	Intervention (*n* = 30)	Control (*n* = 22)	*p*
Age (years)	68.08 ± 10.44	66.67 ± 10.30	70.00 ± 10.57	0.224
Height (cm)	168.93 ± 7.39	168.33 ± 8.28	169.74 ± 6.06	0.770
Weight (kg)	65.75 ± 7.33	64.53 ± 7.95	67.42 ± 6.17	0.075
BMI (kg/m^2^)	23.06 ± 2.29	22.81 ± 2.59	23.40 ± 1.80	0.363
Skeletal muscle mass (kg)	26.58 ± 3.64	26.15 ± 3.98	27.16 ± 3.11	0.663
SBP (mmHg)	128.92 ± 12.81	130.00 ± 12.05	127.45 ± 13.94	0.485
DBP (mmHg)	67.23 ± 13.41	71.60 ± 12.66	61.27 ± 12.29	0.011
Calf circumference, left (cm)	36.15 ± 2.39	35.67 ± 2.66	36.81 ± 1.83	0.089
Calf circumference, right (cm)	35.84 ± 2.42	35.69 ± 2.83	36.05 ± 1.76	0.602
Male, *n* (%)	48 (92.3)	28 (93.3)	20 (90.9)	1.000
Alcohol consumption, *n* (%)	12 (23.08)	10 (33.33)	2 (9.09)	0.051
Smoking, *n* (%)	12 (23.08)	10 (33.33)	2 (9.09)	0.051
Hypertension, *n* (%)	30 (57.69)	18 (60.00)	12 (54.55)	0.078
Diabetes, *n* (%)	18 (34.62)	8 (26.67)	10 (45.45)	0.239
Hyperlipidemia, *n* (%)	24 (46.15)	12 (40.00)	12 (54.55)	0.400

Significance was defined as *p* < 0.05. BMI, body mass index; SBP, systolic blood pressure; DBP, diastolic blood pressure.

**Table 2:** Comparison of physical and cognitive function parameters between the groups before and after 12 weeks.

Variables	Groups	Pre	Post	*F*	*p*
Grip strength (kg)	Intervention	30.80 ± 5.87	32.41 ± 5.16 *	0.718	0.401
	Control	28.06 ± 5.45	30.45 ± 5.85 **		
Gait speed (m/s)	Intervention	1.09 ± 0.18	1.14 ± 0.16 *	12.615	<0.001
	Control	0.99 ± 0.17	0.92 ± 0.22 *		
6MWT (m)	Intervention	405.70 ± 87.86	448.00 ± 69.07 ***	10.345	0.002
	Control	369.80 ± 84.39	361.00 ± 76.94		
FTSS (sec)	Intervention	10.65 ± 1.98	10.26 ± 1.78	0.454	0.504
	Control	12.71 ± 3.09	12.72 ± 3.00		
MVPA (min/day)	Intervention	47.40 ± 49.25	46.80 ± 53.94	2.763	0.103
	Control	35.91 ± 42.78	24.59 ± 27.40		
WHOQOL (points)	Intervention	58.09 ± 8.35	56.91 ± 12.78	0.063	0.803
	Control	62.01 ± 9.44	60.11 ± 12.22		
MMSE (points)	Intervention	27.80 ± 2.14	27.87 ± 2.10	9.397	0.003
	Control	27.18 ± 1.89	28.45 ± 1.65 ***		
SGDS (points)	Intervention	5.33 ± 2.62	5.33 ± 3.32	0.661	0.420
	Control	4.73 ± 3.47	4.18 ± 3.51		

Significant difference between “pre” and “post”: * *p* < 0.05; ** *p* < 0.01; *** *p* < 0.001. 6MWT, 6 min walk test; FTSS, five times sit-to-stand; MVPA, Moderate-to-Vigorous Physical Activity; WHOQOL, World Health Organization quality-of-life assessment; MMSE, Mini-Mental State Examination; SGDS, Short-Form Geriatric Depression Scale.

## Data Availability

Data are available from the author upon reasonable request.

## Supplementary Materials

The following supporting information can be downloaded at https://www.mdpi.com/article/10.3390/life16030441/s1. Figure S1: Importance ranking of measured variables in the random forest model.

## Abbreviations

The following abbreviations are used in this manuscript:

6MWT	6 min walk test
ASO	arteriosclerosis obliterans
BMI	body mass index
FTSS	five times sit-to-stand
ICT	information and communication technology
MMSE	Mini-Mental State Examination
MVPA	moderate-to-vigorous physical activity
PAD	peripheral artery disease
SGDS	Short-Form Geriatric Depression Scale
WHOQOL	World Health Organization quality-of-life assessment
