# Supplementary figures and images for: Effects of an ICT-Based Wearable Intervention on Physical Function in Arteriosclerosis Obliterans: A 12-Week Study
Source: Life (Basel). 2026 Mar 9;16(3):441. doi: 10.3390/life16030441 (PMC13028079; doi:10.3390/life16030441)

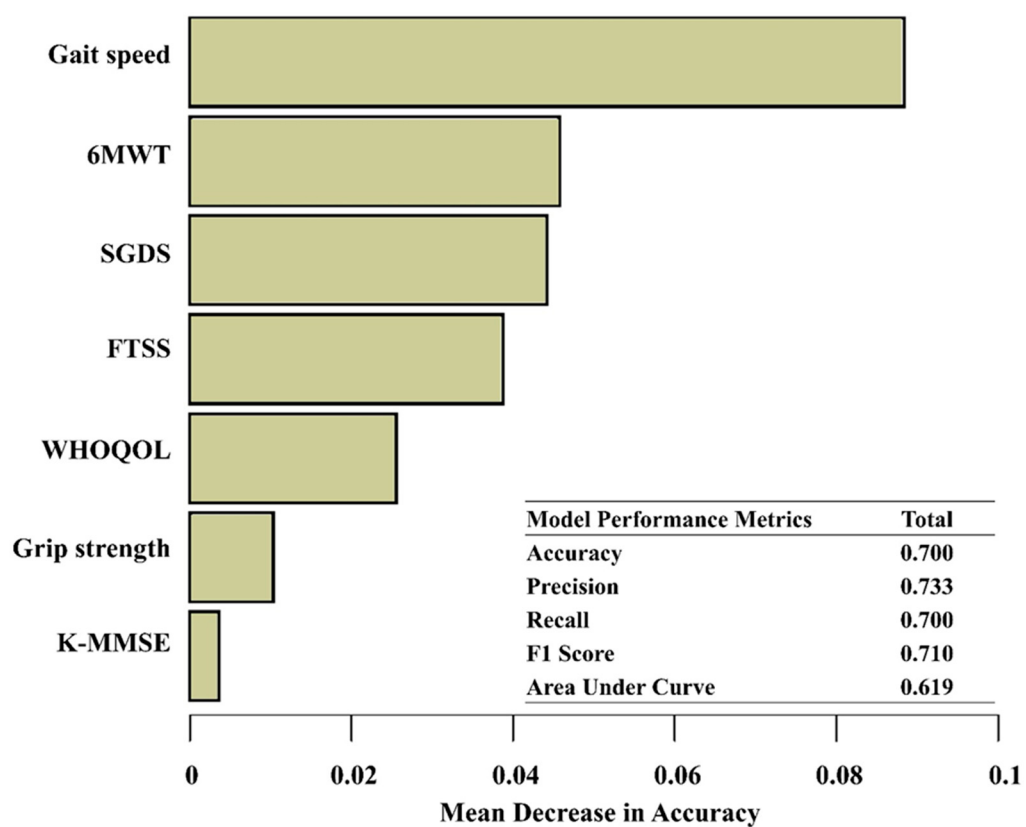

Supplementary Figure S1. Importance ranking of measured variables in the random forest model.

Supplement: Supplementary file 1 [file life-16-00441-s001.zip › life-4156519-supplementary.pdf]
